# ZC3H18 regulates alternative splicing and related genes in cervical cancer

**DOI:** 10.3389/fgene.2025.1621238

**Published:** 2025-09-09

**Authors:** Wenjuan Chen, Chenying Liu, Siyi Li, Xingyun Xie, Dan Hu, Yaobin Lin

**Affiliations:** ^1^ Department of Radiotherapy, Gynecology, Clinical Oncology School of Fujian Medical University, Fujian Cancer Hospital, Fuzhou, China; ^2^ Department of Pathology, Clinical Oncology School of Fujian Medical University, Fujian Cancer Hospital, Fuzhou, China; ^3^ Department of Radiation Oncology, Fujian Medical University Union Hospital, Fujian Key Laboratory of Intelligent Imaging and Precision Radiotherapy for Tumors (Fujian Medical University), Clinical Research Center for Radiology and Radiotherapy of Fujian Province (Digestive, Hematological and Breast Malignancies), Fuzhou, Fujian, China

**Keywords:** alternative splicing events, RNA-binding proteins, ZC3H18, cervical cancer, genes

## Abstract

**Introduction:**

Alternative splicing (AS) and RNA-binding proteins (RBPs) have been implicated in various diseases. However, a comprehensive understanding of their role in RNA metabolism, progression, and metastasis in cervical cancer remains elusive. This study aimed to identify the potential effect of zinc finger CCCH-type containing 18 (ZC3H18) in cervical cancer.

**Methods:**

The Gene Expression Omnibus (GEO) dataset (GSE94427) was used to analyze the expression level of ZC3H18 in HeLa cells and its regulated alternative splicing events (ASEs). The Cancer Genome Atlas (TCGA) cervical cancer dataset and in vitro experiments were used for verification. The signaling pathways and functions of ZC3H18-regulated ASEs were investigated through enrichment analysis.

**Results:**

Knockdown of ZC3H18 in HeLa cells increased the expression of 106 genes but decreased the expression of 226 genes. ZC3H18 was found to be involved in the regulation of 1,830 ASEs. The AS genes were enriched in cervical cancer-related signaling pathways. Validation using 39 cervical cancer samples from the TCGA database showed that 20 cases had low ZC3H18 expression and 19 had high expression. By integrating GEO and TCGA datasets along with in vitro experiments, 18 ASEs with consistent changes were identified.

**Discussion:**

This study demonstrated that ZC3H18 extensively regulates AS of cancer-associated pathways in HeLa cells and cervical cancer tissues. The identification of ZC3H18-regulated ASEs may provide potential targets for cervical cancer treatment.

## Introduction

Cervical cancer is a major gynecological issue in developing and less developed countries (Sung et al., 2021). Infection with a high-risk human papillomavirus (HPV) is the most prevalent cause of cervical cancer (Eklund et al., 2018). Despite significant advancements in early detection and treatment modalities, approximately 30% of patients develop recurrence (Adiga et al., 2021). Patients who relapse usually have a worse prognosis and a higher mortality rate. Therefore, identification of molecular markers and therapeutic targets is significant to improve the therapeutic efficacy of cervical cancer.

In the human genome, alternative splicing (AS) has a significant effect on the complexity of biological functions (Modrek and Lee, 2002). Through alternative splicing mechanisms, a single pre-mRNA transcript can give rise to diverse mRNA splice isoforms. Consequently, a single gene has the capacity to generate multiple distinct protein variants, each potentially playing unique functional roles. In the context of neoplasms, aberrant splicing events are prevalent, often resulting from mutations in splicing cis-regulatory elements or dysregulation of splicing factor expression and function (Anczuków and Krainer, 2016; Song et al., 2018). Aberrantly spliced mRNA transcripts can cause tumor suppressors or oncogene activators to lose their function (Matlin et al., 2005; Sultan et al., 2008). This abnormal regulation of AS, which is involved in various tumor processes, including angiogenesis, apoptosis, migration, and radiochemotherapy sensitivity (Schwerk and Schulze-Osthoff, 2005; Wang et al., 2016; Sheng et al., 2018; Liu et al., 2022; Mabeta and Steenkamp, 2022), has emerged as a novel therapeutic target and biomarker for cancer treatment (Zhao et al., 2017; Urbanski et al., 2018; Peng et al., 2022). Recently, AS has been found to control the development of cervical cancer via the MIL1RAP–NF-κB–CD47 axis (Liu et al., 2018). Che et al. (2023) also demonstrated that the serine/arginine-rich splicing factor 3 (SRSF3), which is highly expressed in cervical cancer, modulates the alternative splicing of exon 12 in the transcriptional cofactor DEAD-box helicase 5 (DDX5). This splicing alteration leads to an increased production of the pro-oncogenic splice variant DDX5-L and a reduction in the levels of the tumor-suppressive splice variant DDX5-S. Consequently, these changes promote the proliferation, migration, and invasion of cervical cancer CaSki cells (Che et al., 2023). Therefore, AS is an interesting therapeutic target in cervical cancer treatment.

RNA-binding proteins (RBPs), which control cell function by interacting with RNA, play significant roles in the post-transcriptional gene expression regulation (Huang et al., 2021). RBPs are involved in different aspects of RNA, such as editing, splicing, transport, metabolism, translation, and maintenance of RNA intracellular localization (Müller-McNicoll and Neugebauer, 2014; Hentze et al., 2018). In recent years, a high level of RBP expression has been observed in cervical cancers associated with HPV infection. Furthermore, they participate in the development of the disease (Huang et al., 2021; Zheng et al., 2022).

Zinc finger CCCH-type containing 18 (ZC3H18), an RBP, is involved in RNA metabolism, participating in transcription activated by the nuclear factor kappa-B (NF-κB) and post-translational modifications (Müller-McNicoll and Neugebauer, 2014; Liu et al., 2018). It plays an essential role in linking RNA metabolism and transcription. Reduced ZC3H18 expression may be beneficial in patients with advanced-stage serous ovarian cancer treated with PARP inhibitors and platinum therapy (Kanakkanthara et al., 2019). However, the mechanism by which ZC3H18 functions in cervical cancer remains unclear.

We hypothesized that the abnormal expression of ZC3H18 in cervical tissue regulates the variable shear of pre-mRNA and influences the development of cervical cancer. This study aimed to investigate the effect of ZC3H18 on regulating gene expression and AS in cervical cancer and HeLa cells using public datasets and *in vitro* experiments and help identify the novel targeted therapies to improve the therapeutic efficacy.

## Materials and methods

### Acquisition of RNA-seq data from the GEO and TCGA datasets

To examine the expression of ZC3H18 and the regulation of AS in HeLa cells and in patients with cervical cancer, RNA-seq data were obtained from the Gene Expression Omnibus (GEO) (GSE94427; https://www.ncbi.nlm.nih.gov/geo) (Giacometti et al., 2017) and The Cancer Genome Atlas (TCGA) ((http://xena.ucsc.edu) datasets. Splice junction data in BED format for cervical cancer samples from TCGA were downloaded from the Genomic Data Commons portal (https://gdc.cancer.gov/) for the identification of variable alternative splicing events (ASEs) (Kahles et al., 2018). The overview of datasets used in this study is presented in Additional file 1. The GEO dataset contained three HeLa cell samples of siRNA targeting ZC3H18 mRNA (siZC3H18 groups) and five control HeLa cell samples [siRNA-targeting firefly luciferase (FFL) mRNA, siFFL groups]. The TCGA database contains clinical data from 39 cases of cervical cancer and RNA-seq data from tumor and adjacent tissues.

### Genetic analysis with respect to differential expression

Pure reads were compared with the human GRch38 genome via HISAT2 (Kim et al., 2019). Using clearly mapped reads, the number of exon fragments per base (FPKM) of mapped fragments and reads per gene were calculated to estimate gene expression levels. DESeq2, software specifically designed for analyzing differentially expressed genes (DEGs), was used for the analysis (Love et al., 2014). The input data used for DEG analysis is the read count. To determine whether there was a differential expression of a gene, we analyzed the results using false discovery rate (FDR <0.05) and fold change (FC ≥ 2 or ≤0.5).

### AS analysis

ASEs and regulatory alternative splicing events (RASEs) are defined and quantified using the ABLas pipeline (Jin et al., 2017; Xia et al., 2017). In short, ABLas identified 10 distinct ASEs by analyzing splice junction reads. These included alternative 5′ and 3′ splice sites (A5SS and A3SS), mutually exclusive exons (MXEs), exon skipping (ES), intron retention (IR), and mutually exclusive 5′ and 3′ untranslated regions (5pMXE and 3pMXE). Additionally, the detection covered cassette exons and complex events combining A3SS with ES or A5SS with ES. Statistical significance of sample pairs was assessed using Fisher’s exact test. After normalizing the reads by RPM (reads per million) of each gene in the samples, an in-house script (sogen) was used for the visualization of next-generation sequencing data and genomic annotations.

Selectively spliced reads and their change ratios were calculated and compared between samples. The RASE detection threshold was defined as a ratio of ≥0.2 with *p* ≤ 0.05. Using Student’s t-test, the meaning of the change in the ASE ratio was assessed during the repeated comparison procedure.

### Enrichment analysis of functions

The KOBAS 2.0 server was used to predict and calculate functional classes of DEGs based on Gene Ontology (GO) analysis and the Kyoto Encyclopedia of Genes and Genomes (KEGG) pathways (Xie et al., 2011). The enrichment pathways were defined using hypergeometric tests and Benjamini–Hochberg FDR controls (corrected *p*-value <0.05). Moreover, a functional enrichment analysis of the selected genes was conducted using the Reactome pathway (http://reactome.org).

### Cell culture and treatment

The human cervical cancer cell line HeLa is preserved by Fuzhou University (Fuzhou, China). HeLa cells were cultured in DMEM (Shanghai BasalMedia Technologies, China) containing 10% fetal bovine serum (FBS, PAN-Biotech GmbH, Germany) in a 5% CO_2_ incubator at 37 °C.

The siRNA fragments of human ZC3H18 and FFL were chemically synthesized by Fuzhou University (Fuzhou, China). The ZC3H18 siRNA sequence was 5′GGAAUGAAUUGUAGGUUUAdTdT; the control FFL siRNA sequence was CUUACGCUGAGUACUUCGAdTdT.

### Reverse transcription PCR

RNA was isolated from all experimental samples using the TRIzol extraction protocol. First-strand cDNA synthesis was then performed using a commercial reverse transcription kit (Yeasen Biotechnology, China) under the following thermal cycling conditions: an initial 2-min incubation at 42 °C, followed by 5 min at 25 °C, a 30-min extension at 42 °C, enzyme inactivation at 85 °C for 5 min, and a final 10-min hold at 4 °C. The reaction used total RNA as the starting template. The reverse transcription PCR (RT-PCR) assay was conducted using the 2× Rapid Taq Master Mix (Vazyme, China) under the following thermal cycling conditions: initial denaturation at 95 °C for 3 min, followed by 35 cycles of 95 °C for 15 s, 60 °C for 15 s, and 72 °C for 15 s per kilobase. Cycle threshold values were recorded for each reaction well. Primer sequences can be found in Additional file 2.

### Statistical analysis

We additionally performed a principal component analysis (PCA) using the R package to illustrate the clustering of the first two samples. The “pheatmap” package in the R language was used. Student’s t-test was used for paired samples with equal variance (P < 0.05 was considered statistically significant).

## Results

### Differential transcription of siZC3H18 in HeLa cells

**FIGURE 1:**
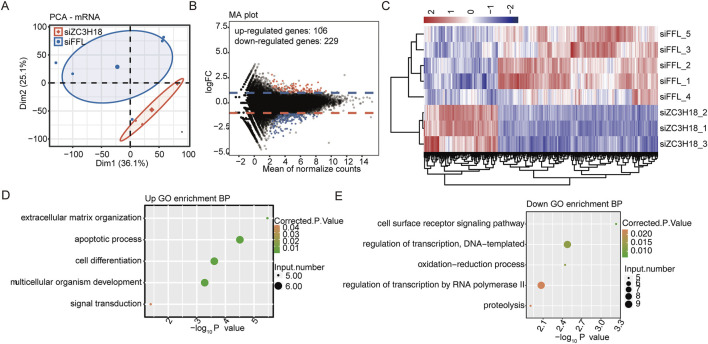
siZC3H18 resulted in transcriptional differences in HeLa cells. **(A)** PCA is the ratio based on all mRNA expressions. Ellipses in the group are the confidence ellipses. **(B)** Detection of ZC3H18-regulated genes on the MA plots. Upregulated genes are marked in red, while downregulated genes are marked in blue. **(C)** Clustering heatmap demonstrating the expression pattern of DEGs among the samples. **(D,E)** Biological process of the up- and downregulated genes, respectively. PCA, principal component analysis; MA plot, M-versus-A plot; DEGs, differentially expressed genes; GO, Gene Ontology.

Clustering analysis was based on the gene expression level of five siFFL samples and three siZC3H18 samples (Figures 1A; Supplementary Figure S1). Three siZC3H18 samples were grouped together, and five siFFL samples were grouped together. Consequently, a clear difference in gene expression was observed between the two groups. The M-versus-A plot indicates that there were 335 differentially expressed genes between siZC3H18 and siFFL, with 106 upregulated genes and 229 downregulated genes (Figure 1B and Additional file 3). Cluster analysis revealed distinct groupings of siZC3H18 and siFFL samples based on DEG expression levels (Figure 1C).

The abovementioned upregulated- or downregulated gene expressions were extracted for functional analysis. These upregulated genes were mostly concentrated in signaling pathways related to extracellular matrix organization, apoptotic process, cell differentiation, and multicellular organism development (Figure 1D and Additional file 4). These downregulated genes were primarily related to the cell surface receptor signaling pathway, DNA template transcription regulation, oxidation–reduction process, and RNA polymerase II transcription regulation (Figure 1E and Additional file 4). Therefore, ZC3H18 may play a significant role in cervical tumorigenesis.

### Identification of abnormal ASEs in HeLa cells

**FIGURE 2:**
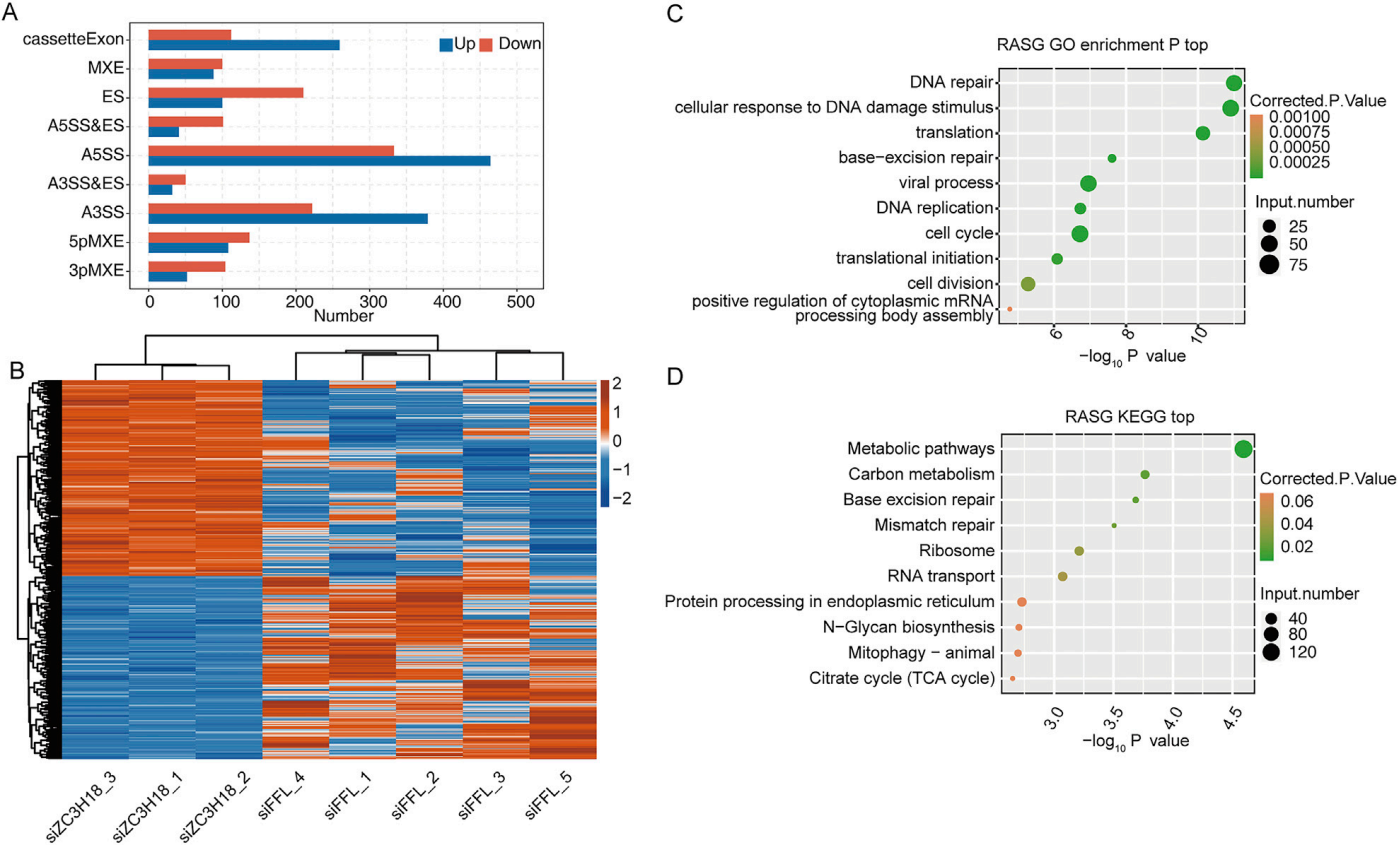
Identification of alternative splicing events regulated by ZC3H18 in the HeLa cell lines. **(A)** Bar plot of the distribution of RASEs between the siZC3H18 and siFFL groups. Blue and red bars display the number of up- and downregulated RASEs in the siZC3H18 group, respectively, compared with those in the siFFL group. **(B)** Heatmap of hierarchical clustering based on the ratio of significant RASEs. **(C,D)** The scatter plots show the most enriched biological process and the KEGG pathway in RASGs, respectively.

According to the analysis of RASEs, the siZC3H18 groups significantly differed in terms of several types of RASEs, such as A5SS, compared with the siFFL groups (Figure 2A and Additional file 5). In total, 1,830 ASEs were regulated by ZC3H18. Clustering analysis revealed that RASEs in the siZC3H18 groups differed from those in the siFFL groups (Figure 2B). To validate the potential functionality of these RASEs, genes for which RASEs have occurred were extracted and subjected to GO and KEGG analyses (Figures 2C,D and Additional files 6–7). GO analysis indicated that the genes undergoing regulated alternative splicing (RASGs) were associated with various biological processes, including DNA replication, response to DNA damage stimulus, translation, viral process, cellular DNA repair, and cell cycle. KEGG pathway analysis demonstrated that RASGs play a role in multiple biological processes, including metabolic regulation, DNA repair mechanisms (specifically base excision and mismatch repair), ribosomal function, RNA translocation, and endoplasmic reticulum-associated protein processing. These findings suggest that ZC3H18 could potentially influence cervical cancer development and therapeutic outcomes by regulating ASEs. The Venn diagram indicated that 19 genes were differentially expressed between the siZC3H18 and siFFL groups and were regulated by ZC3H18-mediated alternative splicing (Supplementary Figure S3A and Additional file 8). These 19 genes were involved in glycine, serine, and threonine metabolisms, steroid hormone biosynthesis, ECM–receptor interaction, axon guidance, and metabolic pathways (Supplementary Figure S3B and Additional file 9).

### Analysis of potential ZC3H18-regulated ASEs in cervical cancer

**FIGURE 3:**
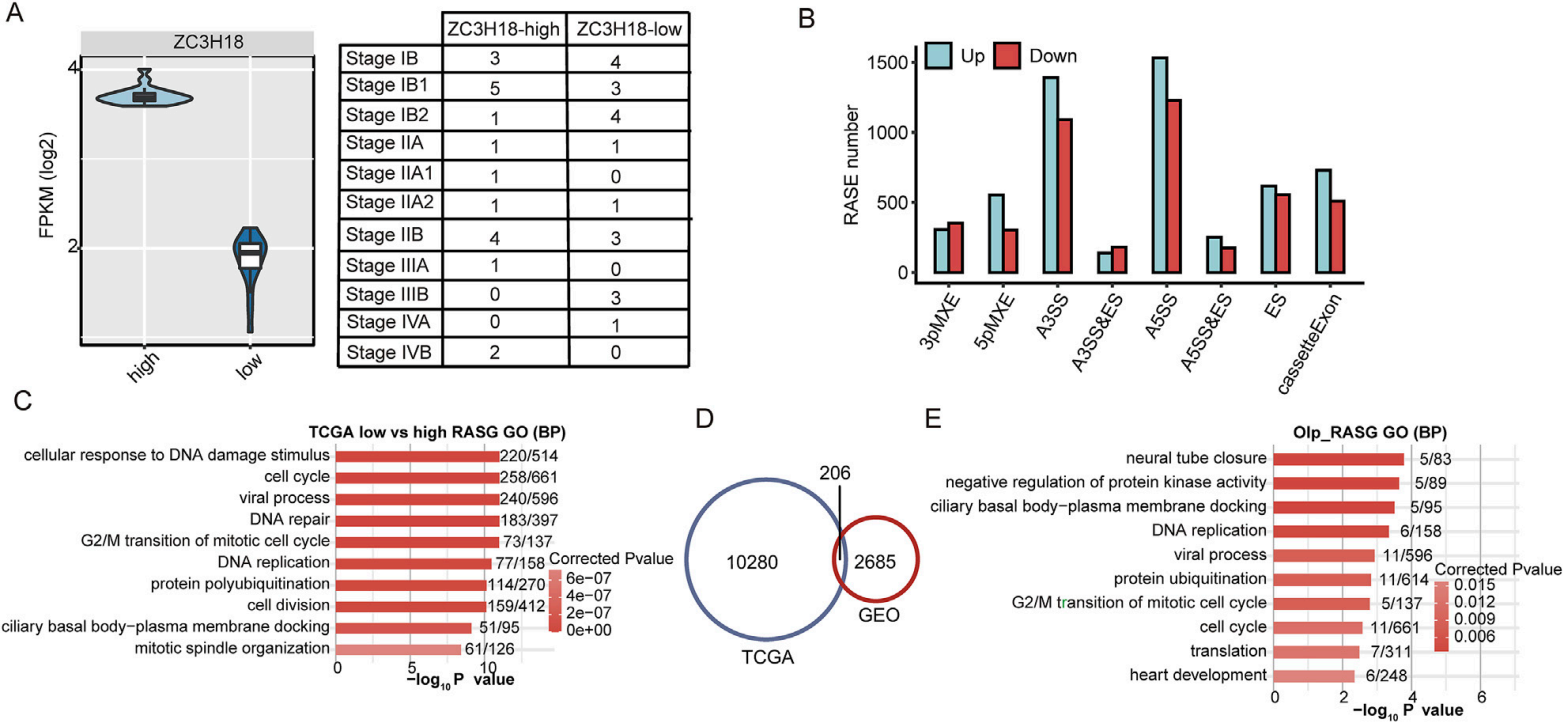
Transcriptome analysis in ZC3H18-mediated alternative splicing. **(A)** Plot shows different expressions of ZC3H18 in the two groups. **(B)** Bar plot shows the distribution of RASEs between low and high expression of ZC3H18. The green and red bars show the number of up- and downregulated RASEs in the low ZC3H18 expression, respectively, compared with those in the high ZC3H18 expression. **(C)** Top 10 GO analysis of biological process in which the RASGs were enriched. **(D)** The RASG intersection was shown in the Venn diagram between the TCGA and GEO databases. **(E)** Top 10 GO biological process analyses in the 206 RASGs.

To identify ZC3H18-regulated RASEs in cervical cancer samples, 39 cases were downloaded from TCGA, of which 19 showed high ZC3H18 expression and 20 showed low ZC3H18 expression (Figure 3A and Additional file 10). The ZC3H18 expression levels were not associated with pathological stages or survival (Figure 3A; Supplementary Figure S2).

We compared the RASE levels between groups with low and high expressions of ZC3H18. Consistent with the results in the GEO database, low ZC3H18 expression increased the ratios of A3SS, A5SS, and cassette exon events (Figure 3B and Additional file 11). The signaling pathways involved in RASGs were also consistent with the GEO results. Furthermore, they were associated with cellular response to DNA damage, cell cycle, viral processes, DNA repair, G2/M transition in the mitotic cell cycle, and DNA replication (Figure 3C and Additional file 12).

The RASGs regulated by ZC3H18 in the GEO and TCGA databases were intersected, resulting in 206 genes (Figure 3D and Additional file 13). GO analysis showed that these genes were involved in multiple functions, including ciliated basal body–plasma membrane docking, negative regulation of protein kinase activity, and DNA replication and mitosis of the cell cycle (Figure 3E and Additional file 14). Therefore, ZC3H18-regulated RASGs may be involved in tumorigenesis and progression in cervical cancer.

### Validation of ZC3H18-regulated ASEs

**FIGURE 4:**
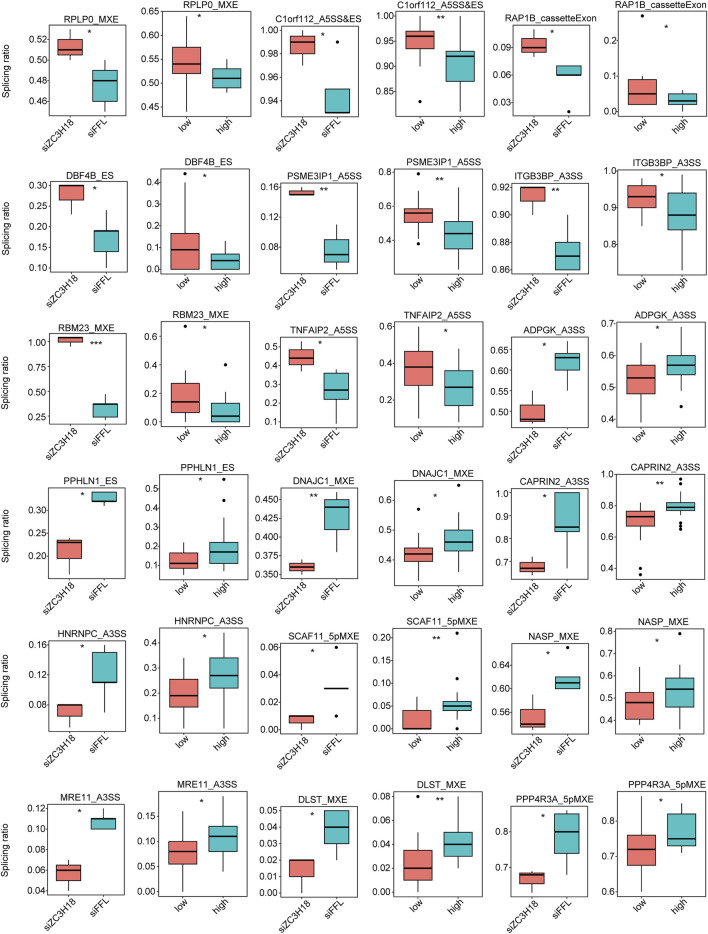
Validation of ZC3H18-regulated alternative splice events in the GEO dataset using TCGA data.

**FIGURE 5:**
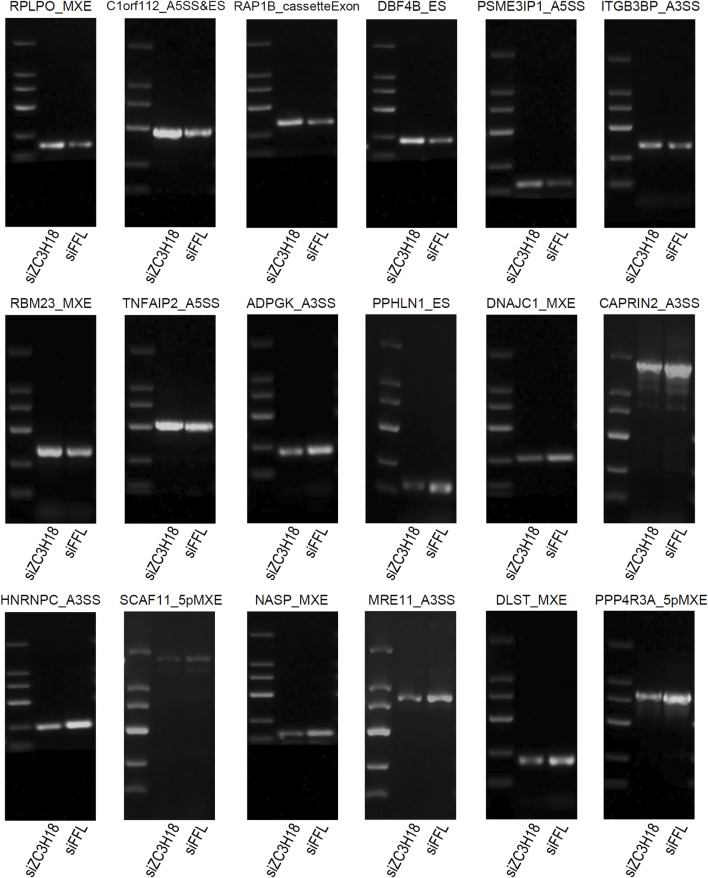
Validation of ZC3H18-regulated alternative splice events in the GEO and TCGA datasets using the *in vitro* experiment.

The RASEs were identified using the GEO dataset and validated using the TCGA dataset and *in vitro* experiments. Eighteen genes had the same type of AS in the two datasets, and the regulatory trend was consistent (Figure 4). Their Integrative Genomics Viewer (IGV) is presented in Additional file 15. In addition, we knocked down the expression of ZC3H18 by siRNA, and *in vitro* experiments also verified that these 18 RASEs were regulated by ZC3H18 (Figure 5). Of these events, 10 (55.56%) RASEs were reduced at low ZC3H18 levels. Therefore, these events (ADPGK_A3SS, PPHLN1_ES, DNAJC1_MXE, CAPRIN2_A3SS, HNRNPC_A3SS, SCAF11_5pMXE, NASP_MXE, MRE11_A3SS, DLST_MXE, and PPP4R3A_5pMXE) were positively regulated by ZC3H18. The remaining eight RASEs were negatively regulated by ZC3H18.

## Discussion

Cervical cancer ranks among the leading cancers in low-income nations (Sung et al., 2021). Despite significant progress in treating cervical cancer, such as the development of advanced radiotherapy techniques and targeted therapies, there has been a limited improvement in treatment efficacy. Unfortunately, the overall 5-year survival rate is only 17% (Zeng et al., 2018). Hence, novel therapeutic target biomarkers should be identified to improve treatment modes in cervical cancer.

The role of AS in human cancer has garnered considerable attention in recent times. AS generates protein-coding gene diversity and plays a critical role in the development and progression of malignant diseases. Splicing factors, which are actually RBPs and are frequently dysregulated in cancer, commonly govern the process of AS development. KEGG pathway analysis revealed that cervical cancer initiation and development are regulated by RBPs through the mRNA surveillance pathway, RNA degradation, and RNA transport. AS, an important contributor to protein-coding gene diversity, is involved in the development of malignancies. A recently published paper has shown that RBPs are highly expressed in cervical cancer and are also linked to HPV infection (Fay et al., 2009).

ZC3H18 is highly expressed in a wide range of tumor tissues, with higher expression in squamous intraepithelial lesions than in normal cervical tissue. ZC3H18 is an RBP that affects gene expression via several methods. For example, it triggers the activation of the NF-κB transcription factor through an unidentified process (Gewurz et al., 2012) and modulates RNA metabolism by engaging in mRNA splicing and transport (Chi et al., 2014), directing RNA degradation via the exosome (Andersen et al., 2013). However, the role of ZC3H18 in the regulation of AS in cervical cancer remains unclear. In the study, the RNA-seq data of HeLa cells and 39 cervical cancer tissues were integrated to analyze the association between ZC3H18 and AS.

After suppressing ZC3H18 expression in HeLa cells, the expression of 335 genes exhibited changes. Among these genes, 106 showed increased expression, potentially due to the elevated levels of unstable nuclear transcripts resulting from ZC3H18 depletion. These findings align with the role of ZC3H18 in facilitating CBCN-mediated RNA decay mediated by the exosome (Andersen et al., 2013; Giacometti et al., 2017; Iasillo et al., 2017). However, more importantly, the mRNA level decreased (229, 68.36%), which could not be explained by the RNA degradation function of ZC3H18. A prior investigation proposed that these observations could indicate an as-yet-unidentified role of ZC3H18 that is independent of exosome activity (Winczura et al., 2018). Therefore, the function of ZC3H18 can still be explored.

The database results showed that ZC3H18 was significantly involved in the regulation of RASEs in cervical cancer. Among the five AS types (cassette exon, A5SS, A3SS&ES, A3SS, and 3pMXE), the results obtained from the GEO and TCGA databases were consistent. The variation trends in ES, A5SS&ES, and 5pMXE regulated by ZC3H18 differed in the two datasets, which might be related to individual differences among samples. Consistent with previous reports, A5SS and A3SS accounted for the largest proportion of AS events identified in HeLa cells and cervical cancer tumor tissues (Jin et al., 2017; Xia et al., 2017). Changes in the 5′ splicing site of RNA precursor molecules can lead to different exon combinations, which, in turn, produce different transcripts and proteins. Although translation regulation is mediated by sequence-specific elements in the 5′ untranslated region (UTR) and 3′ UTR, most sequences affecting translation are located in the 3′ UTR (Hussey et al., 2011). Levesque et al. (2021) comprehensively explored the role of A3SS in breast cancer and found that it was related to the development of breast cancer. Nevertheless, to our knowledge, comprehensive investigations examining the involvement of A5SS and A3SS in cervical cancer are lacking.

ZC3H18 can regulate the splicing of some genes in clinical samples and HeLa cells. Functional enrichment analysis showed that ZC3H18-related RASGs are involved in several biological processes, including key cancer-related pathways. HPV replicates when its long control region interacts with host cytokines, thereby triggering transcription of the E6 and E7 genes.

Furthermore, by binding to and disabling tumor suppressor proteins, cell cycle proteins, and their associated kinases, E6 and E7 genes control the host cell growth cycle (Syrjänen and Syrjänen, 1999). For example, the HPV E6 gene product interacts with p53, leading to its rapid degradation by intracellular ubiquitin ligases, thereby disrupting the normal functions of p53 in regulating G1 cell cycle arrest, apoptosis, and DNA repair (Thomas et al., 1999). The HPV E7 gene product can bind to the RB protein family and cyclin E, leading to cell cycle disorders (Syrjänen and Syrjänen, 1999). This study found that ZC3H18-regulated RASGs were also involved in the viral process, DNA replication, and cell cycle. Therefore, the close association between ZC3H18 and cervical cancer highlights its potential as a promising diagnostic and therapeutic target.

Based on the research findings from both GEO and TCGA datasets, RASGs linked to ZC3H18 are functionally implicated in DNA repair pathways. Poly ADP-ribose polymerase (PARP), a critical DNA repair enzyme, plays a pivotal role in DNA damage repair and cellular apoptosis (Slade, 2020; Zhu et al., 2020). PARP inhibitors have emerged as essential therapeutic agents in first-line and maintenance treatment for homologous recombination-deficient (HRD) ovarian cancer (Caruso et al., 2025; Papageorgiou et al., 2025). Significant benefits of PARP inhibitors have also been documented in endometrial cancer through multiple studies (Janzen et al., 2013; Ji et al., 2025). Given that cervical cancer cells with high ZC3H18 expression exhibit significant enrichment in DNA repair pathways, PARP inhibitors may potentially enhance disease control rates in this patient subset.

Nevertheless, this study had several limitations. Although we also constructed a HeLa cell line with ZC3H18 knockdown to verify the 18 RASEs, the findings of this study are mainly based on the public data, and additional experiments should be performed to validate the role of ZC3H18 in AS and elucidate the potential mechanisms by which it regulates substrate genes or protein isoforms. In addition, prospective clinical data are required to validate the diagnostic and therapeutic value of ZC3H18 in cervical cancer, given the inherent limitations of this retrospective analysis.

## Conclusion

The current study applied the GEO dataset, TCGA dataset, and *in vitro* experiment to identify the regulation of ZC3H18 on AS in cervical cancer. The results showed that ZC3H18-regulated RASEs were correlated with viral processes, DNA replication, and cell cycles in HeLa cells and clinical samples of cervical cancer. Thorough investigation of ZC3H18-mediated alternative splicing will enable a more precise understanding of the signaling pathways involved in cancer development and may reveal potential therapeutic strategies targeting ZC3H18.

## Data Availability

The datasets presented in this study can be found in online repositories. The names of the repository/repositories and accession number(s) can be found in the article/Supplementary Material.

## Supplementary material

SUPPLEMENTARY FIGURE S1 Expression of the *ZC3H18* gene in the siFFL and siZC3H18 groups of the GEO dataset.

SUPPLEMENTARY FIGURE S2 Influence of high and low expression levels of the *ZC3H18* gene on the overall survival of patients with cervical cancer.

SUPPLEMENTARY FIGURE S3 Identification of alternative splicing events in HeLa cells. **(A)** The Venn diagram illustrates the overlap analysis results between ZC3H18-regulated DEGs and RASGs. **(B)** The bar plot displays the most enriched findings of the 19 overlapping genes in the KEGG pathway.

## Abbreviations

HPV, human papillomavirus; AS, alternative splicing; SRSF3, serine/arginine-rich splicing factor 3; DDX5, DEAD-box helicase 5; RBPs, RNA-binding proteins; ZC3H18, zinc finger CCCH-type containing 18; NF-κB, nuclear factor kappa-B; FFL, firefly luciferase; FPKM, fragments per kilobase of exon per million fragments mapped; DEGs, differentially expressed genes; FC, fold change; FDR, false discovery rate; ASEs, alternative splicing events; RASEs, regulated alternative splicing events; PCA, principal component analysis; RASGs, regulated alternative splicing genes; UTR, untranslated region; IGV, Integrative Genomics Viewer; PARP, poly ADP-ribose polymerase.
