# Supplementary material for: ZC3H18 regulates alternative splicing and related genes in cervical cancer
Source: Front Genet. 2025 Sep 9;16:1621238. doi: 10.3389/fgene.2025.1621238 (PMC12454084; doi:10.3389/fgene.2025.1621238)

RPLP0_MXE


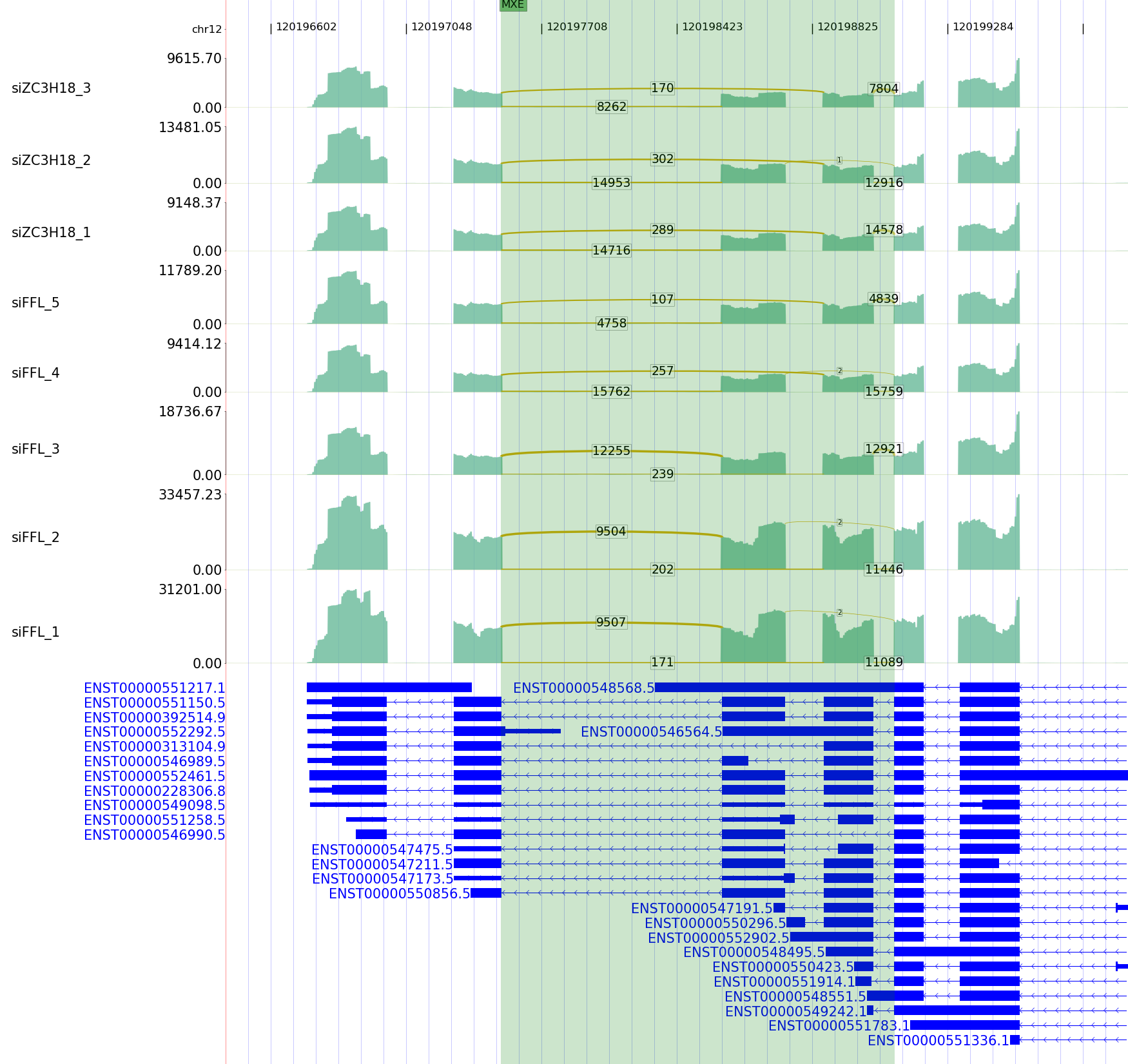


C1orf112_A5SS&ES


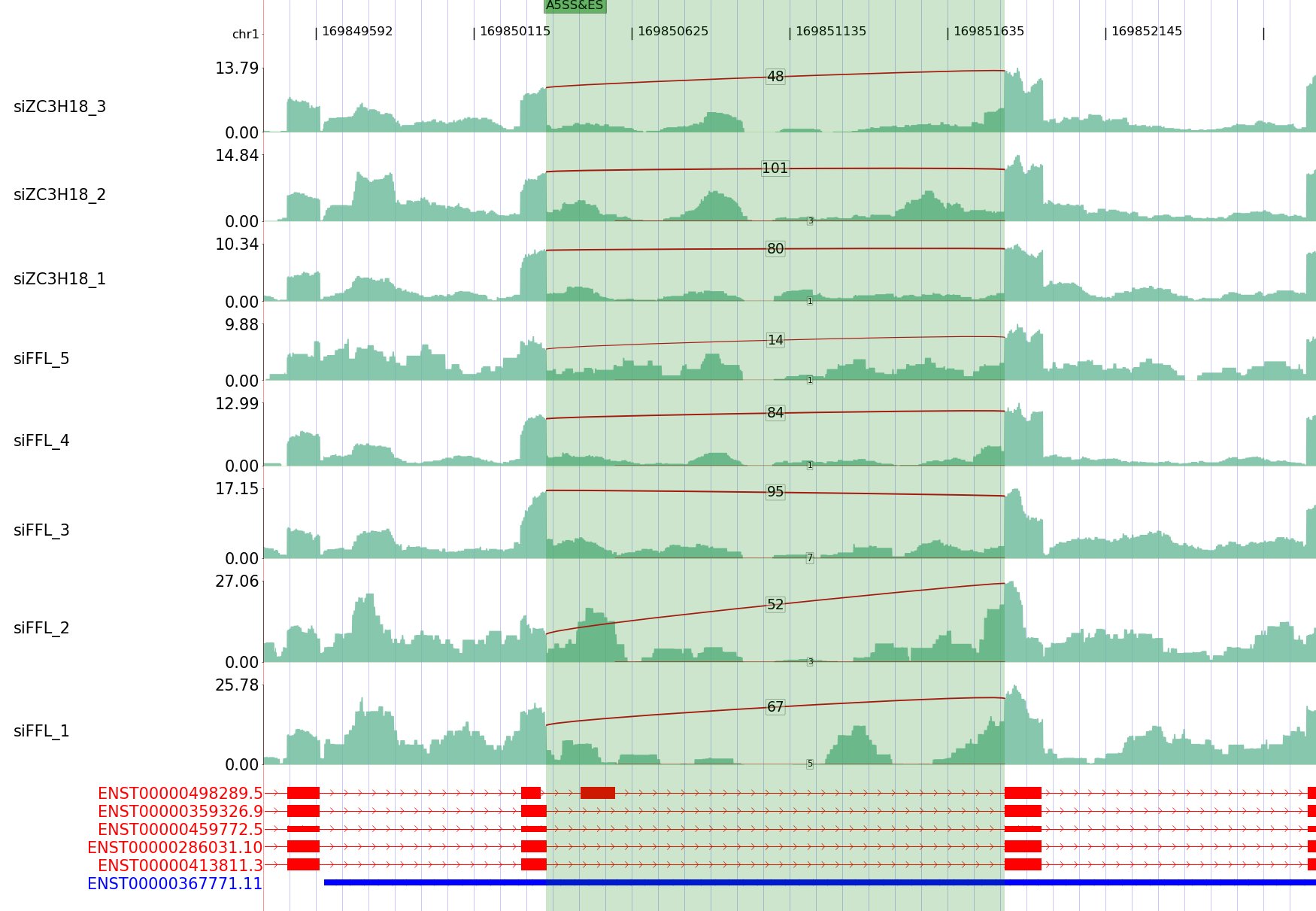


RAP1B_cassetteExon


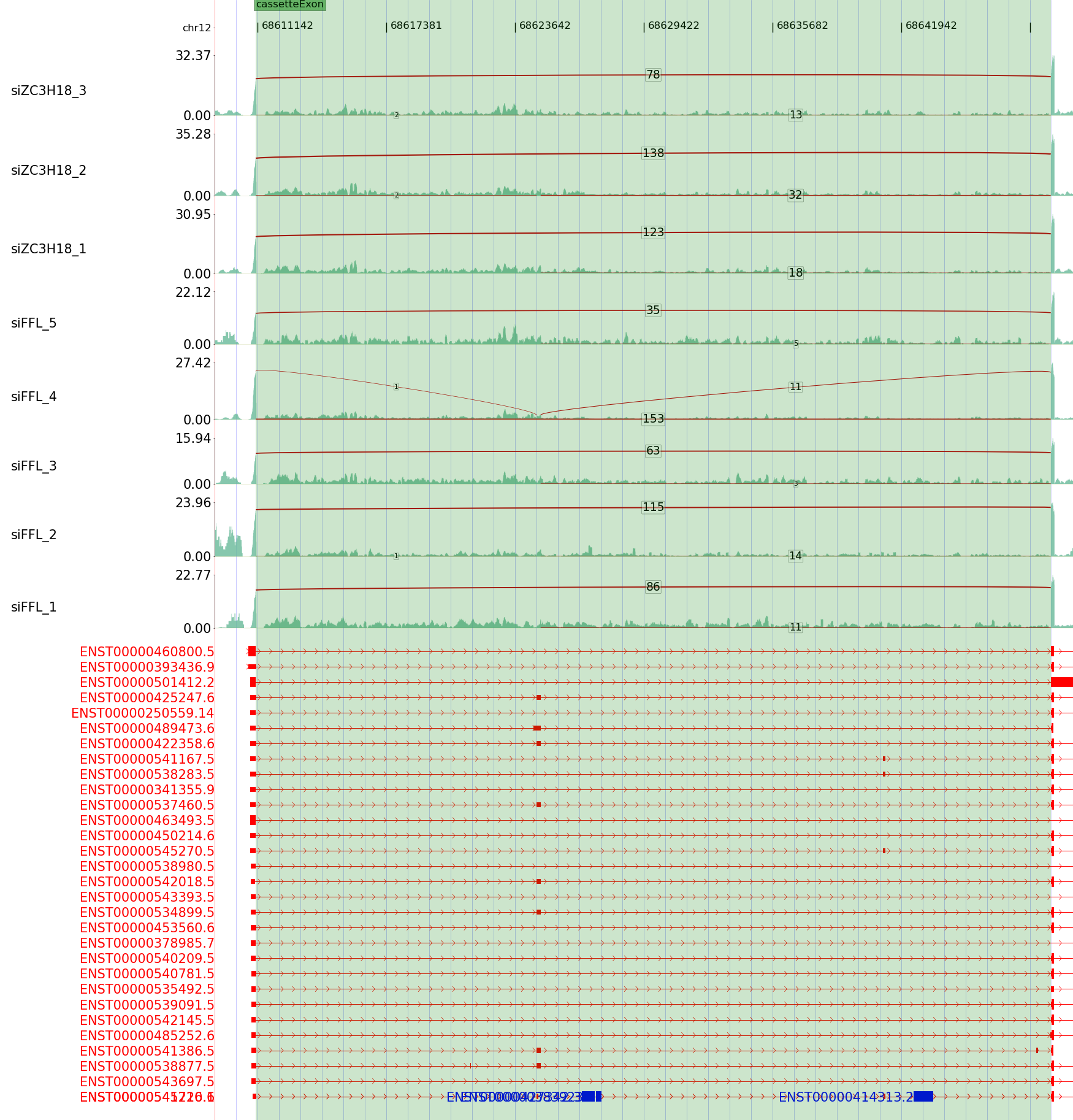


DBF4B_ES


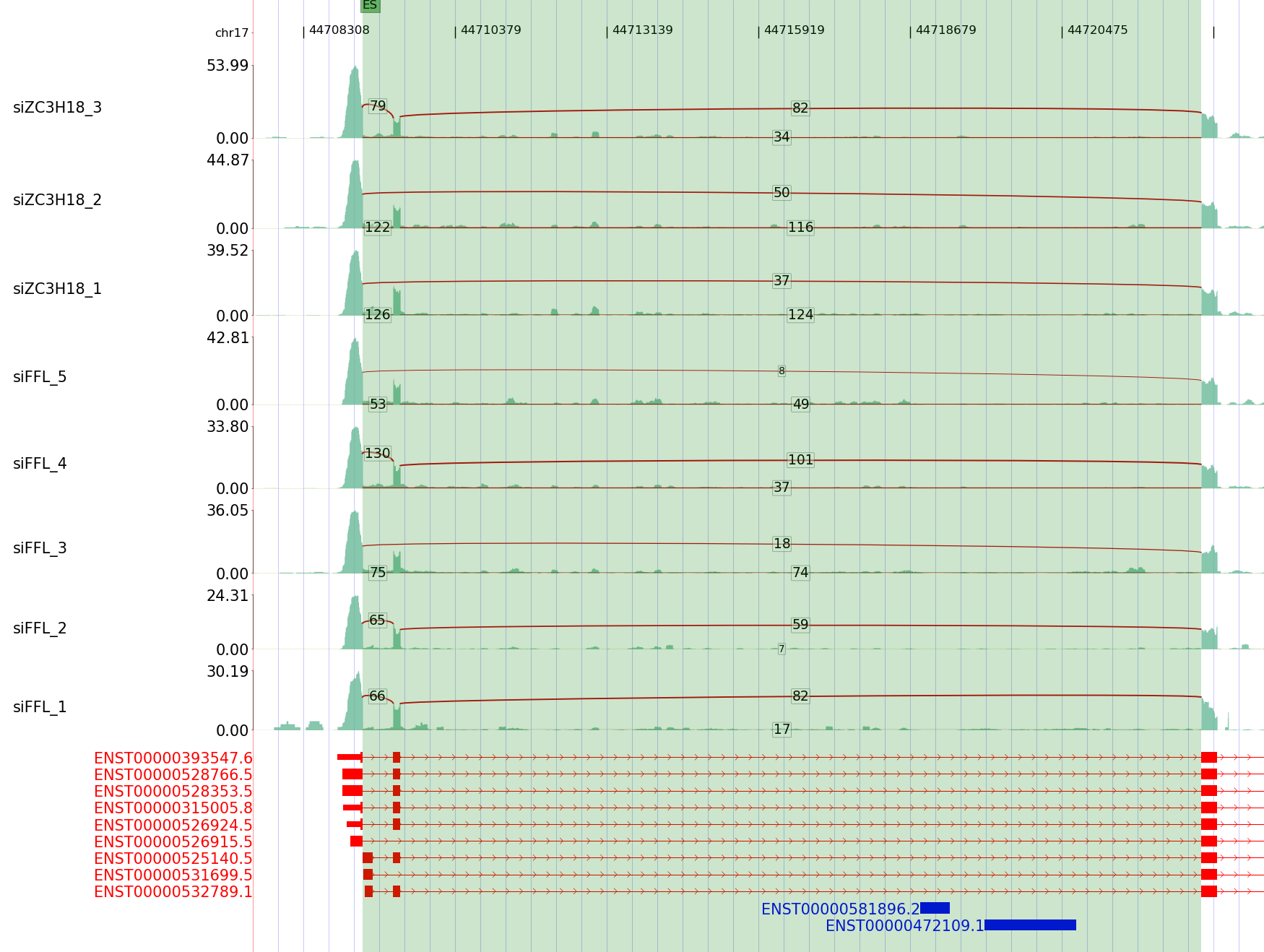


PSME3IP1_A5SS


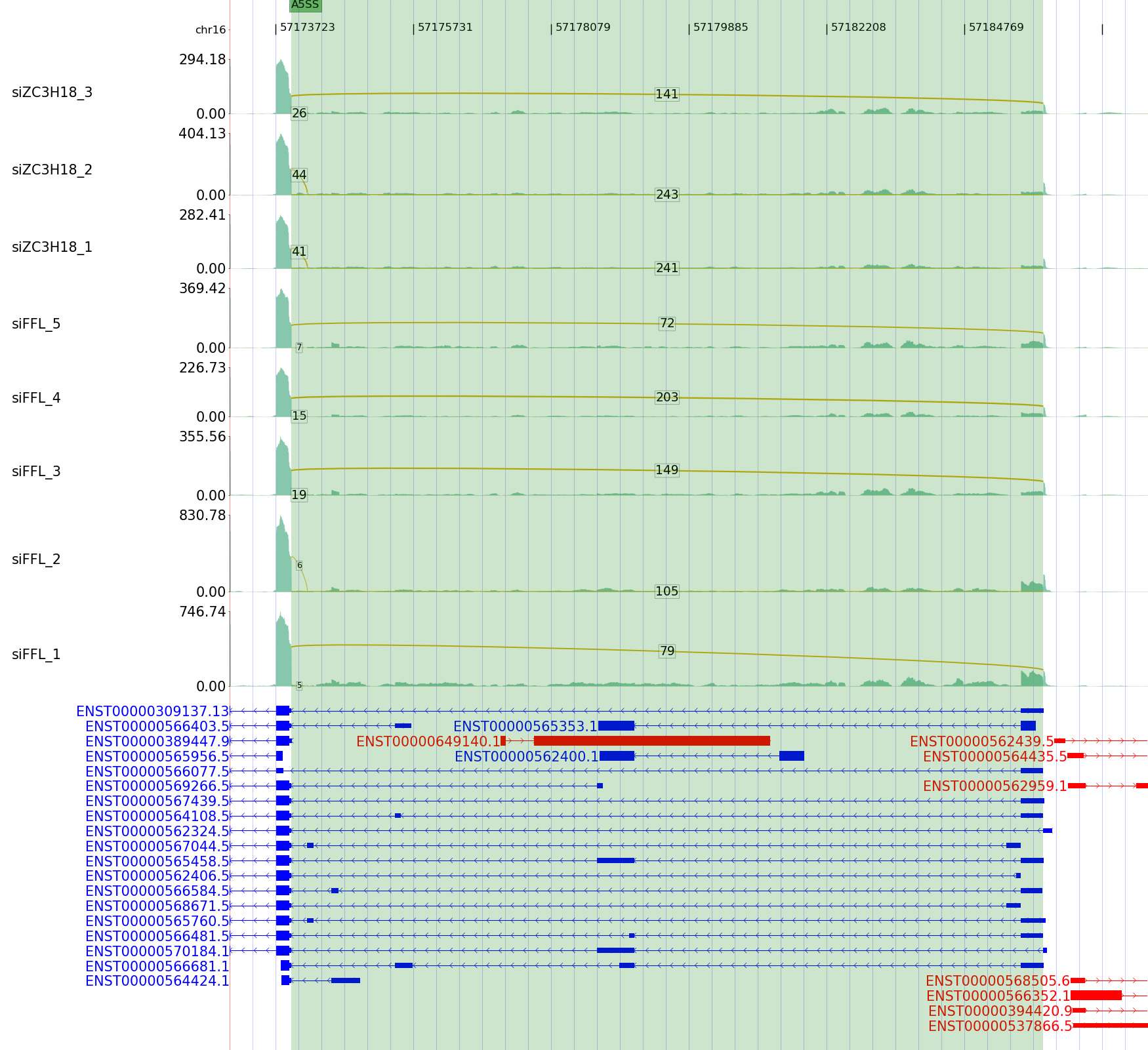


ITGB3BP_A3SS


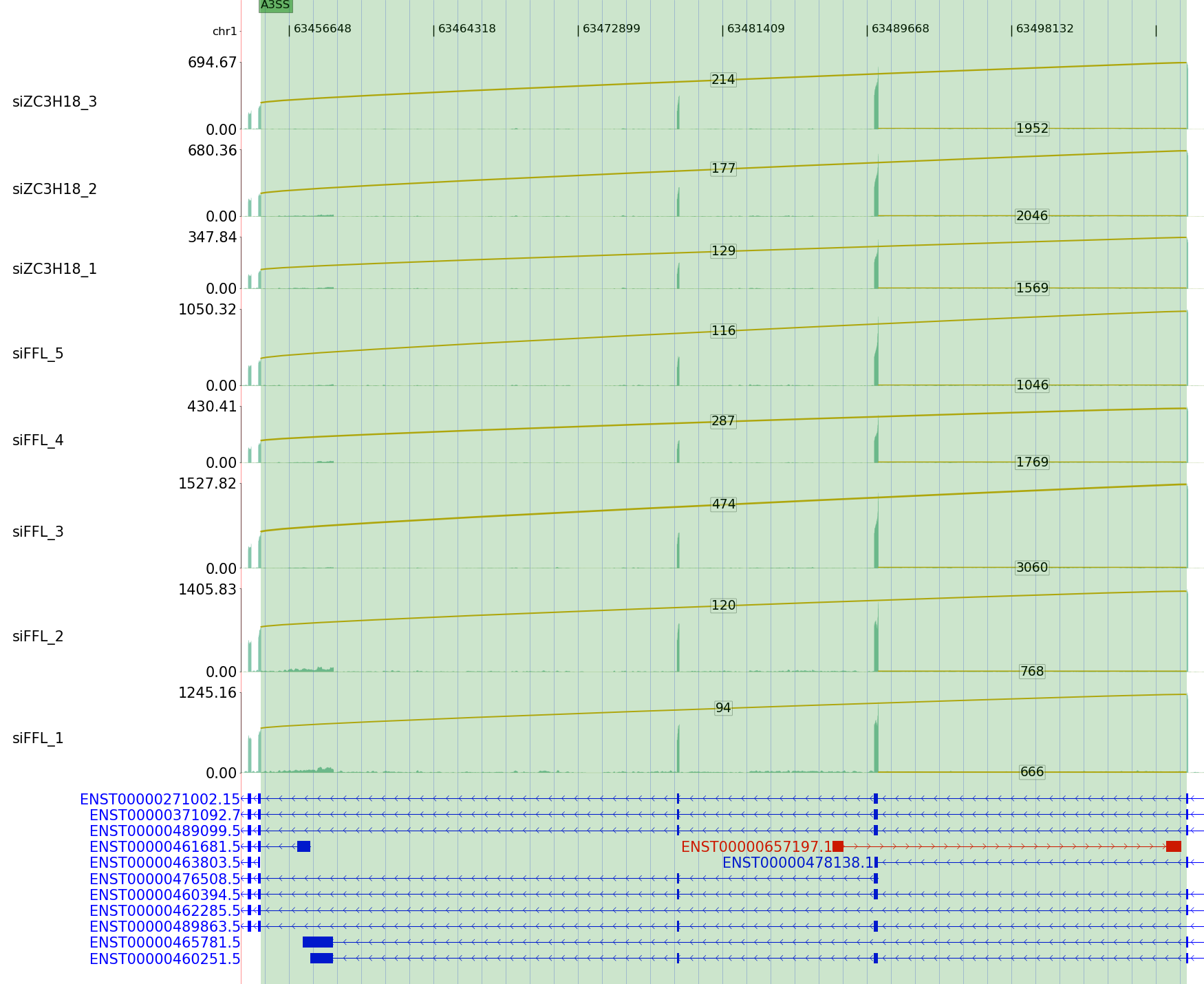


RBM23_MXE


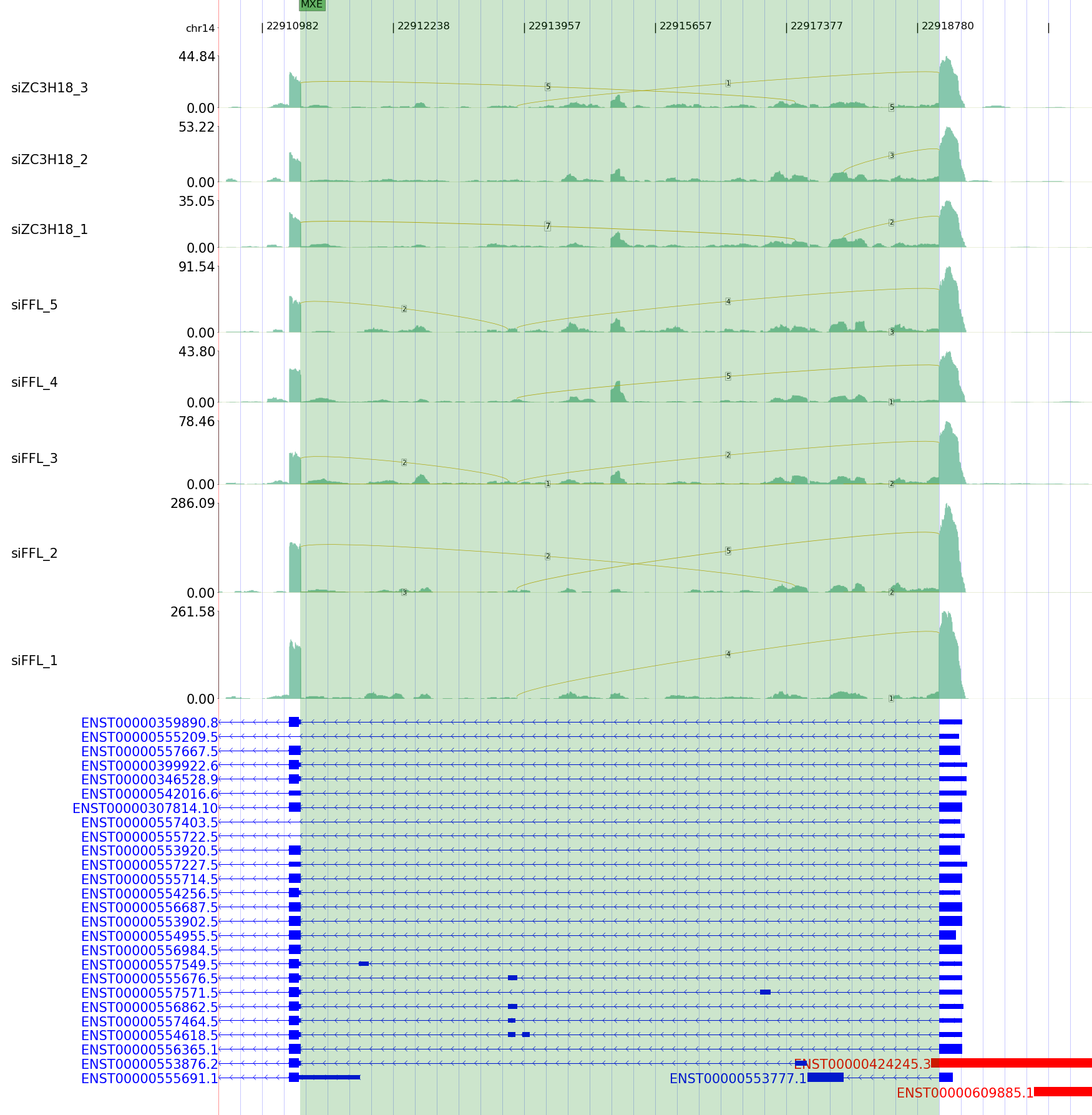


TNFAIP2_A5SS


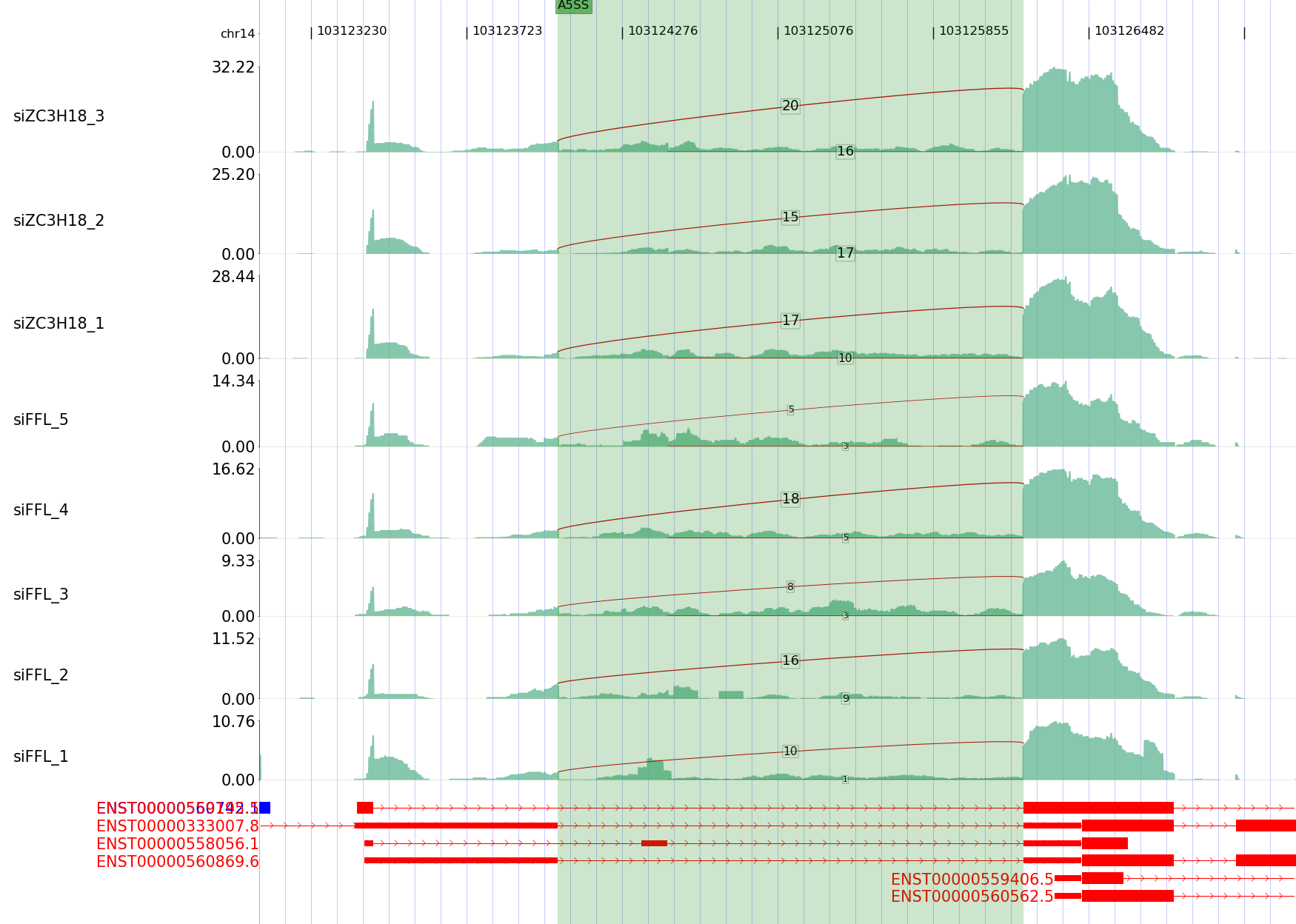


ADPGK_A3SS


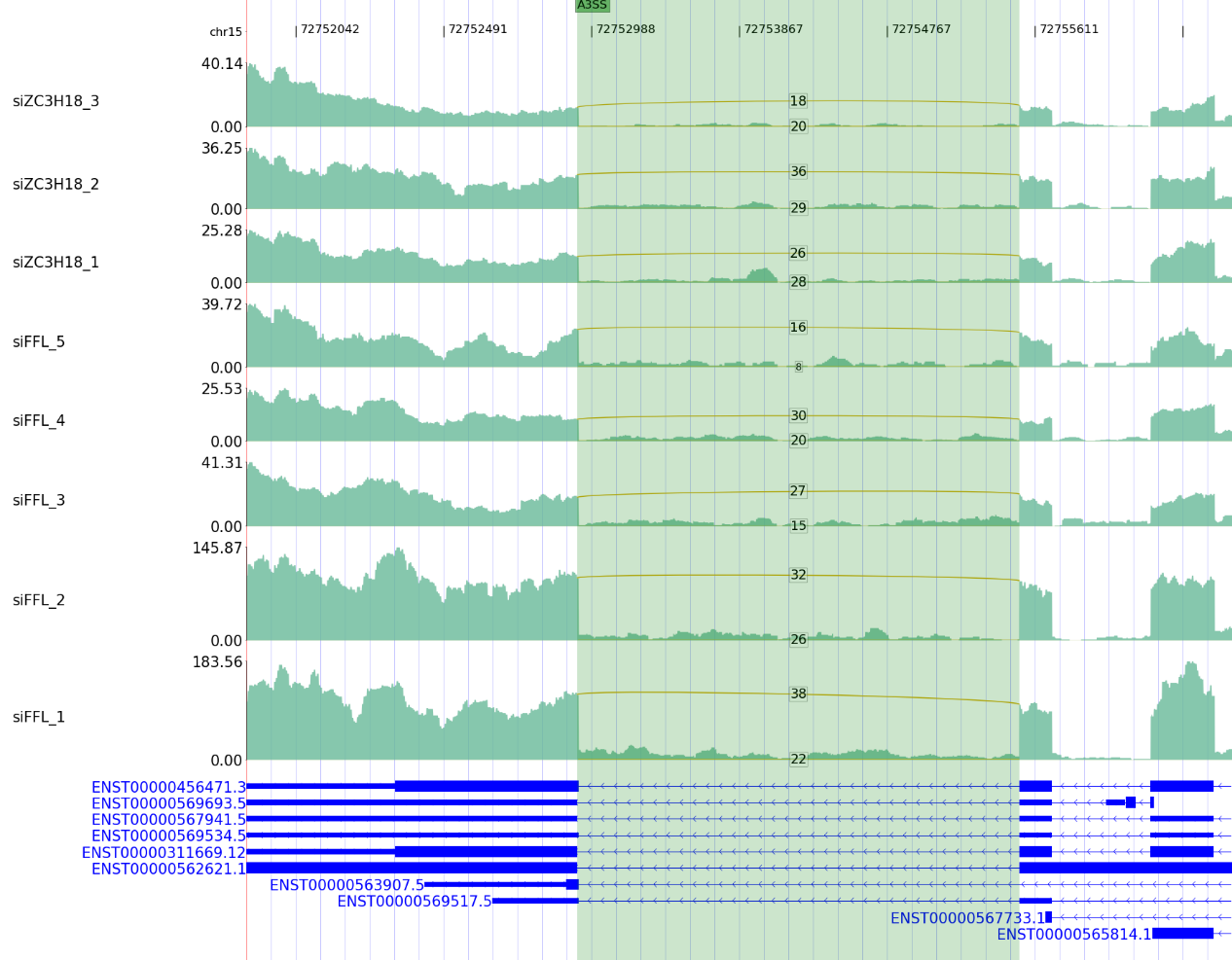


PPHLN1_ES


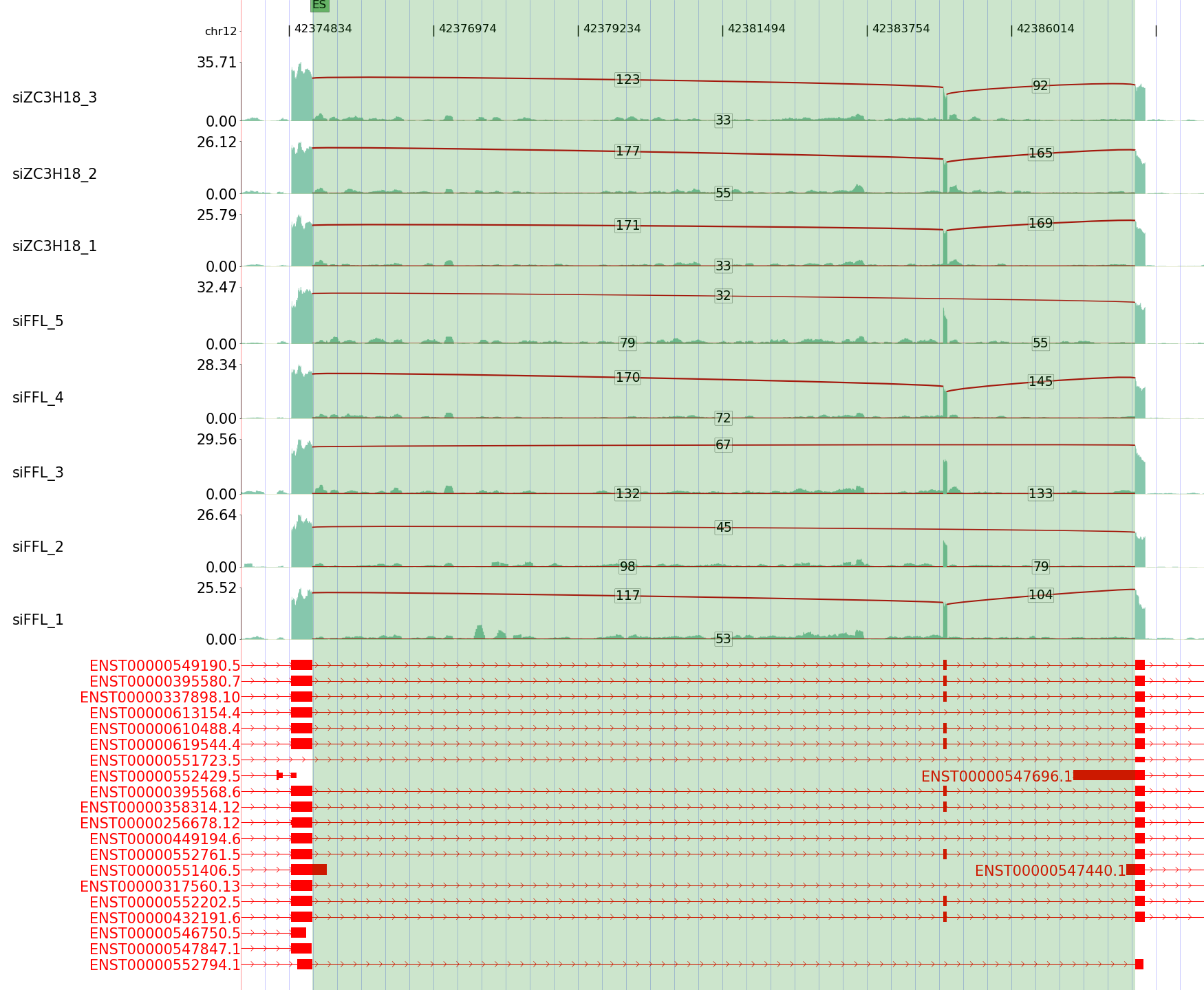


DNAJC1_MXE


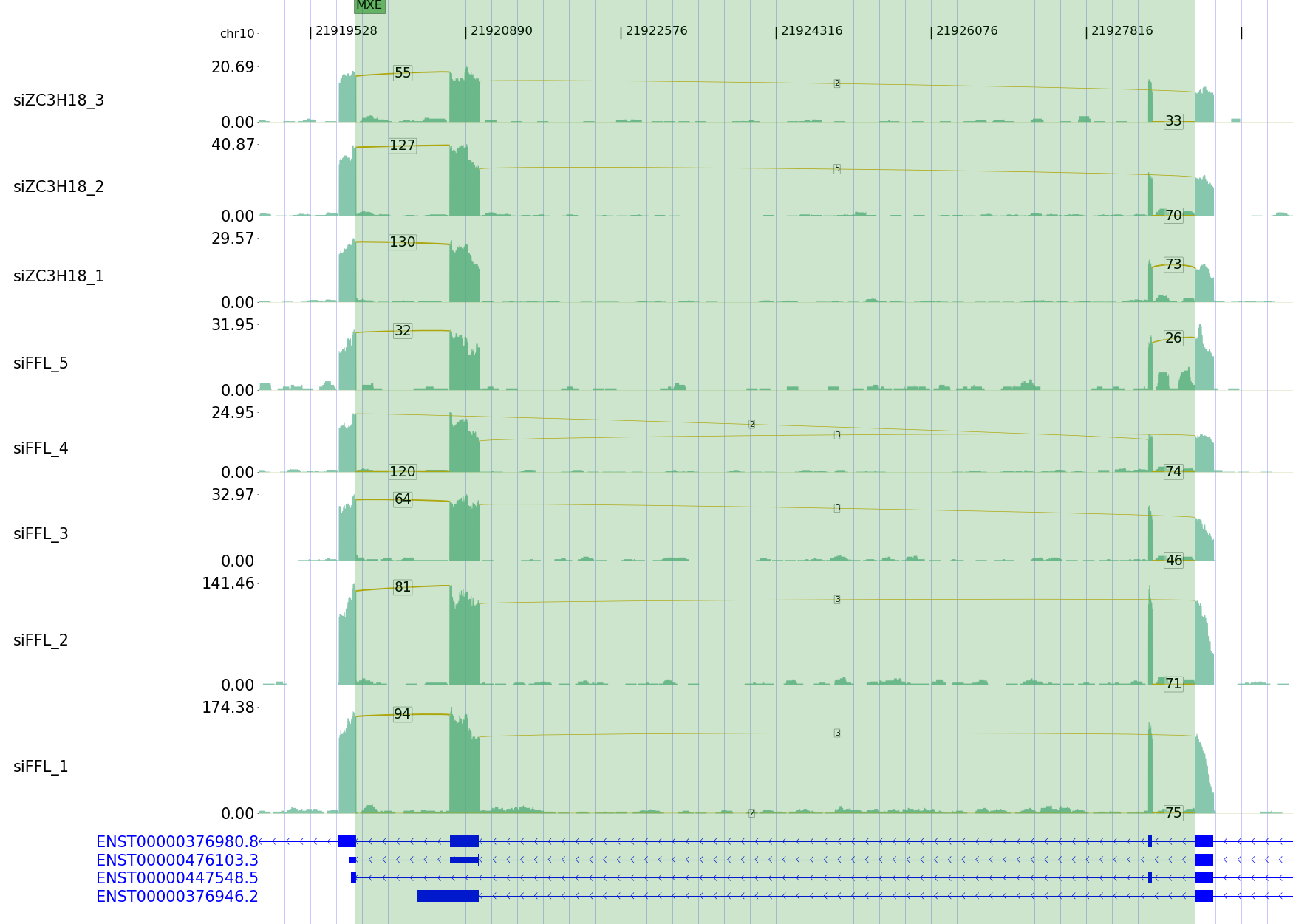


CAPRIN2_A3SS


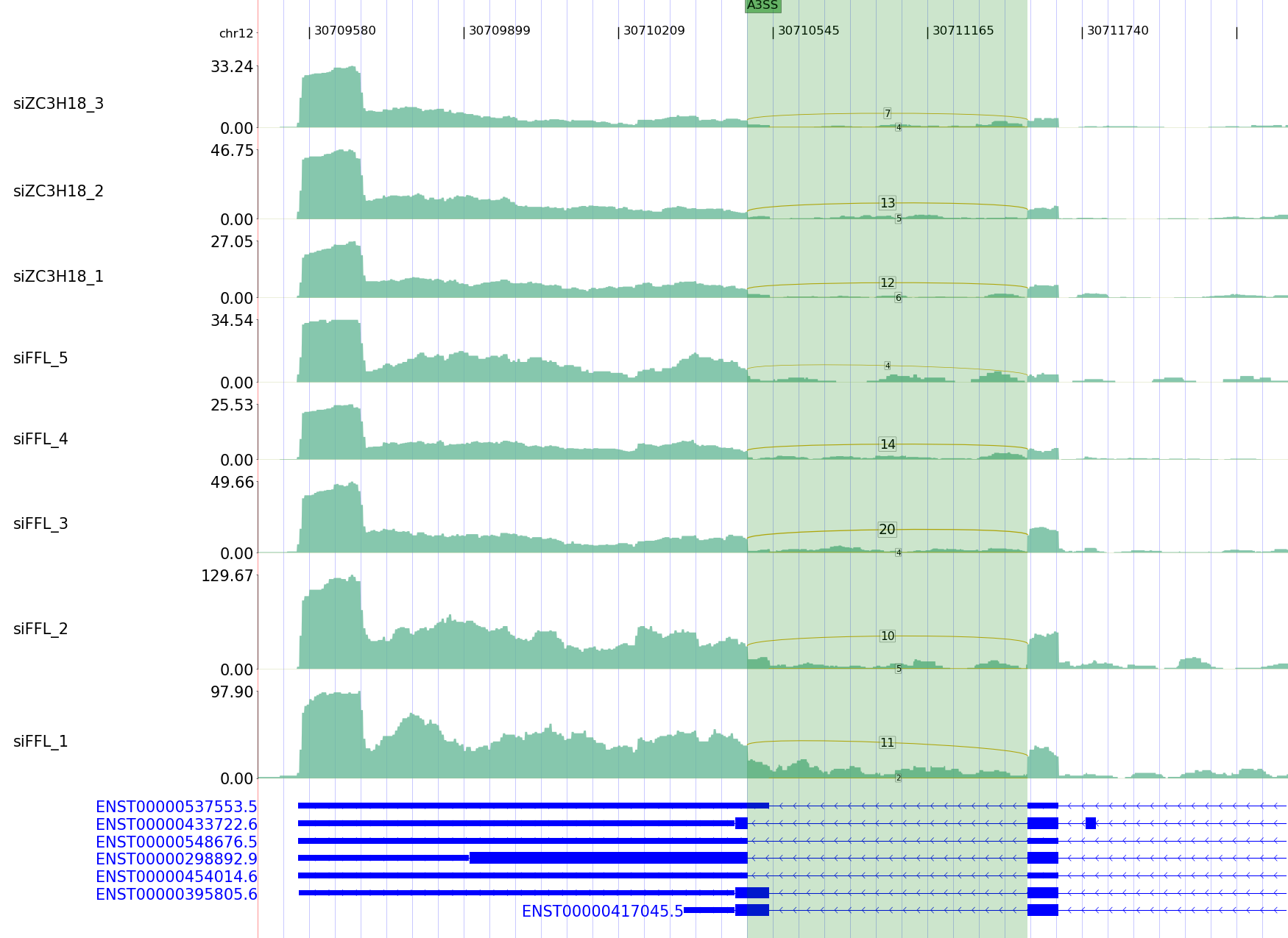


HNRNPC_A3SS


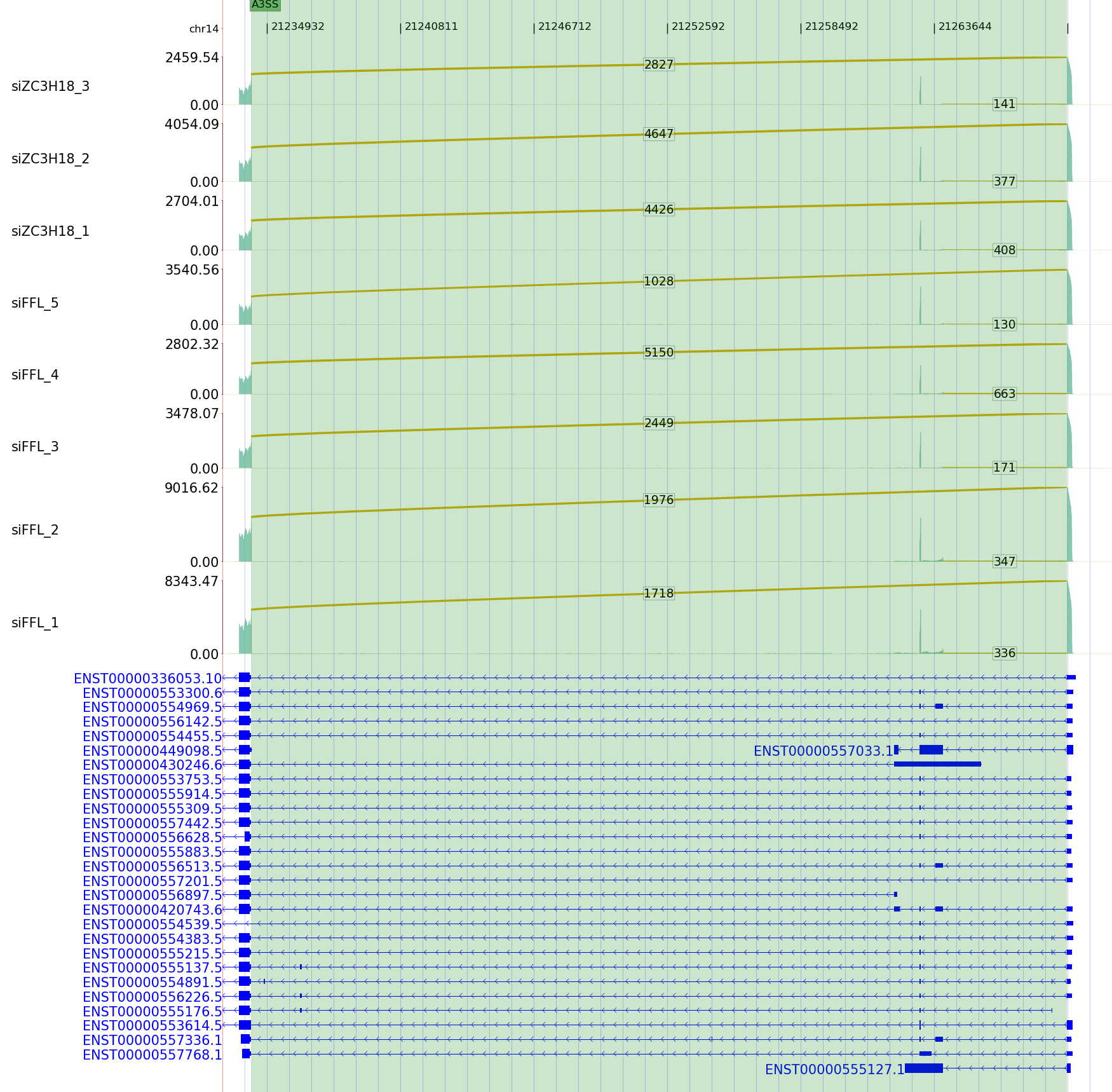


SCAF11_5pMXE


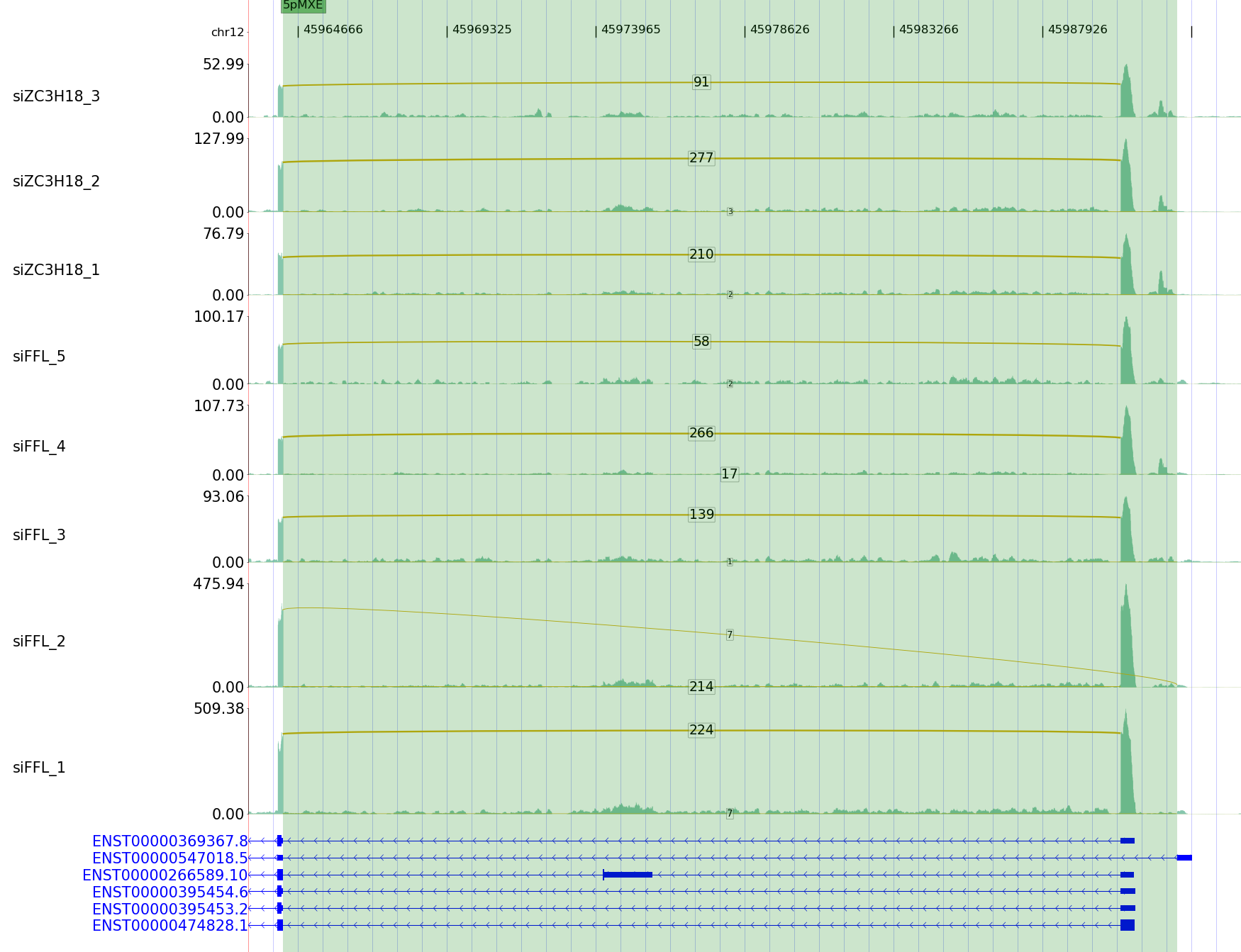


NASP_MXE


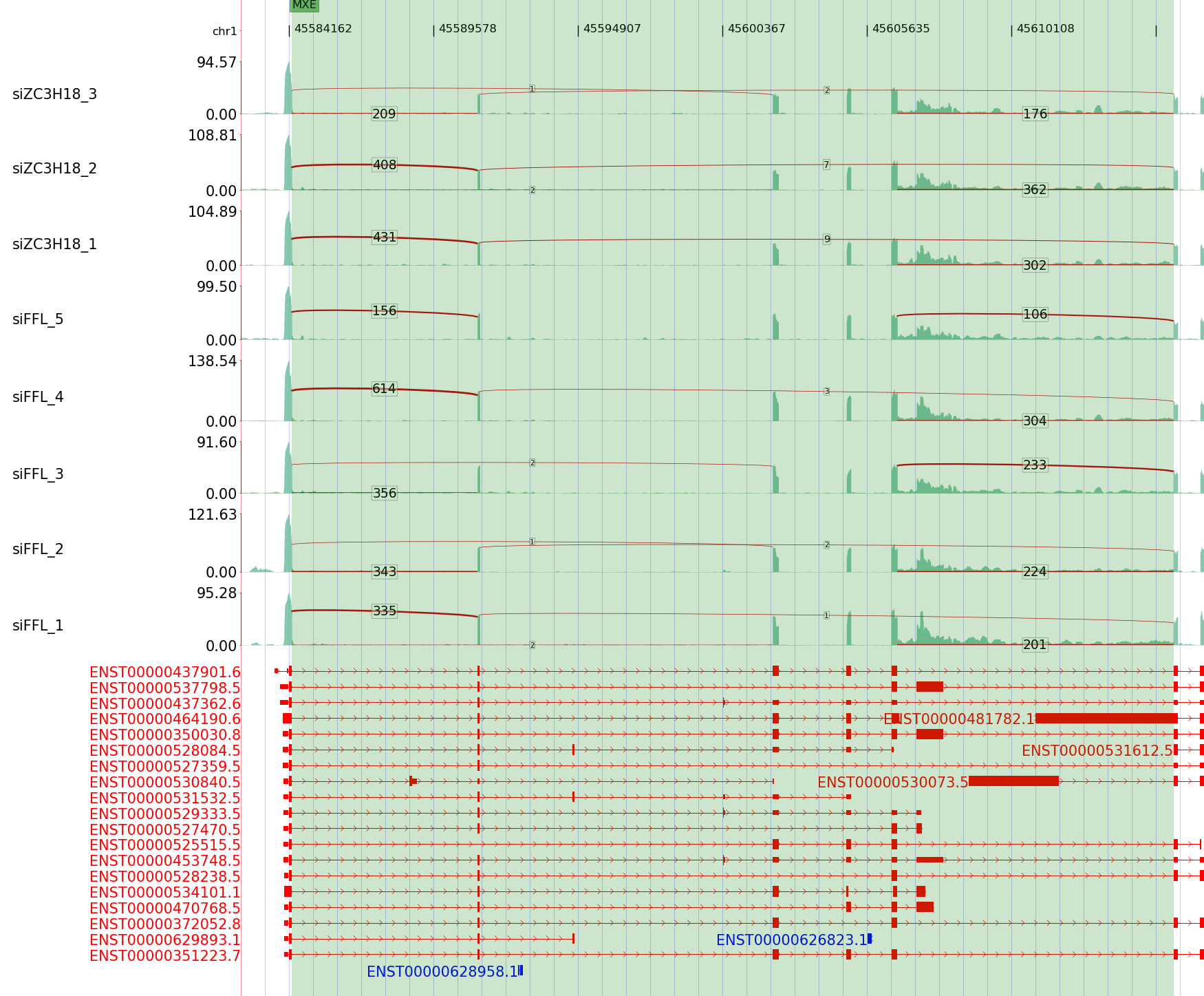


MRE11_A3SS


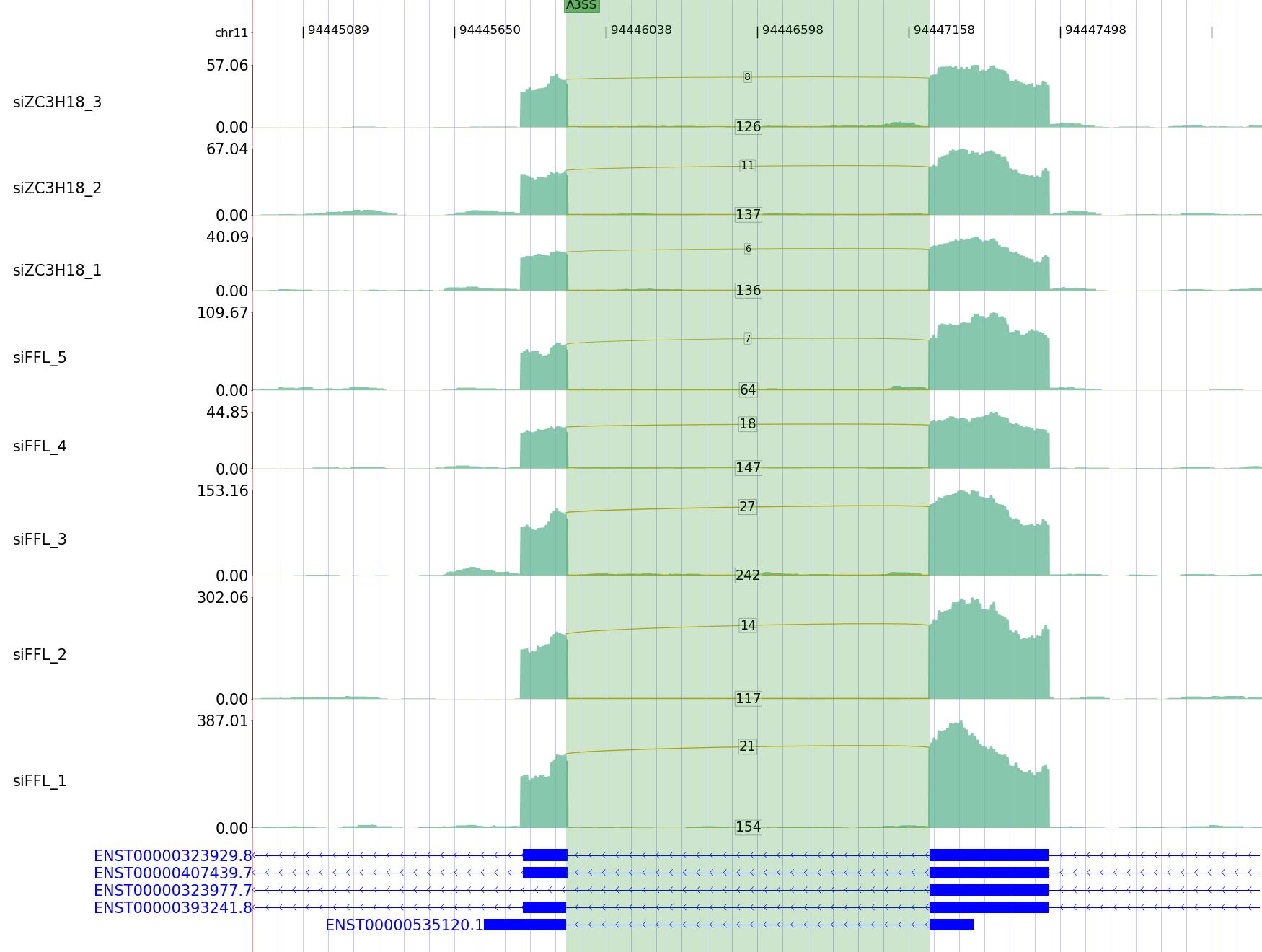


DLST_MXE


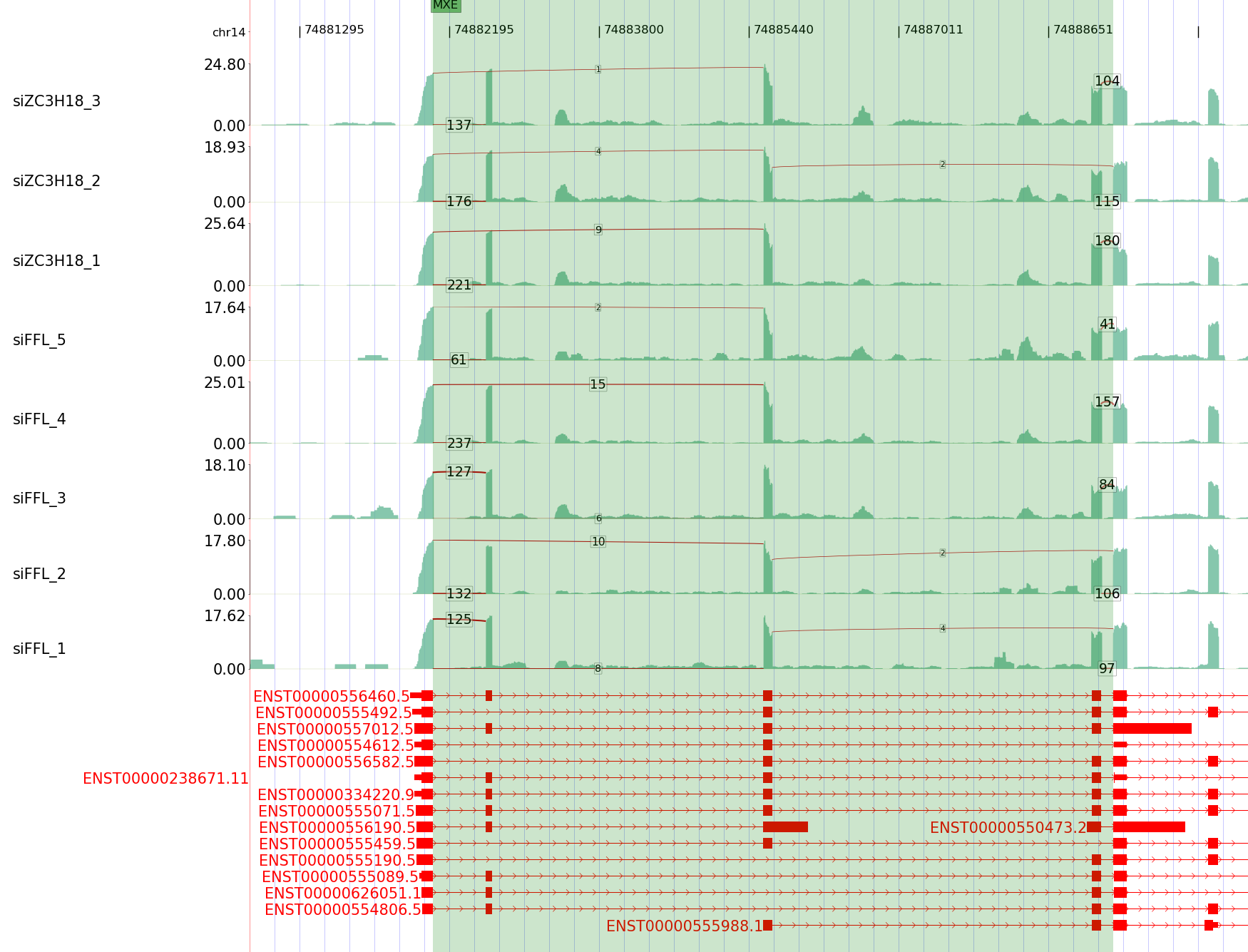


PPP4R3A_5pMXE


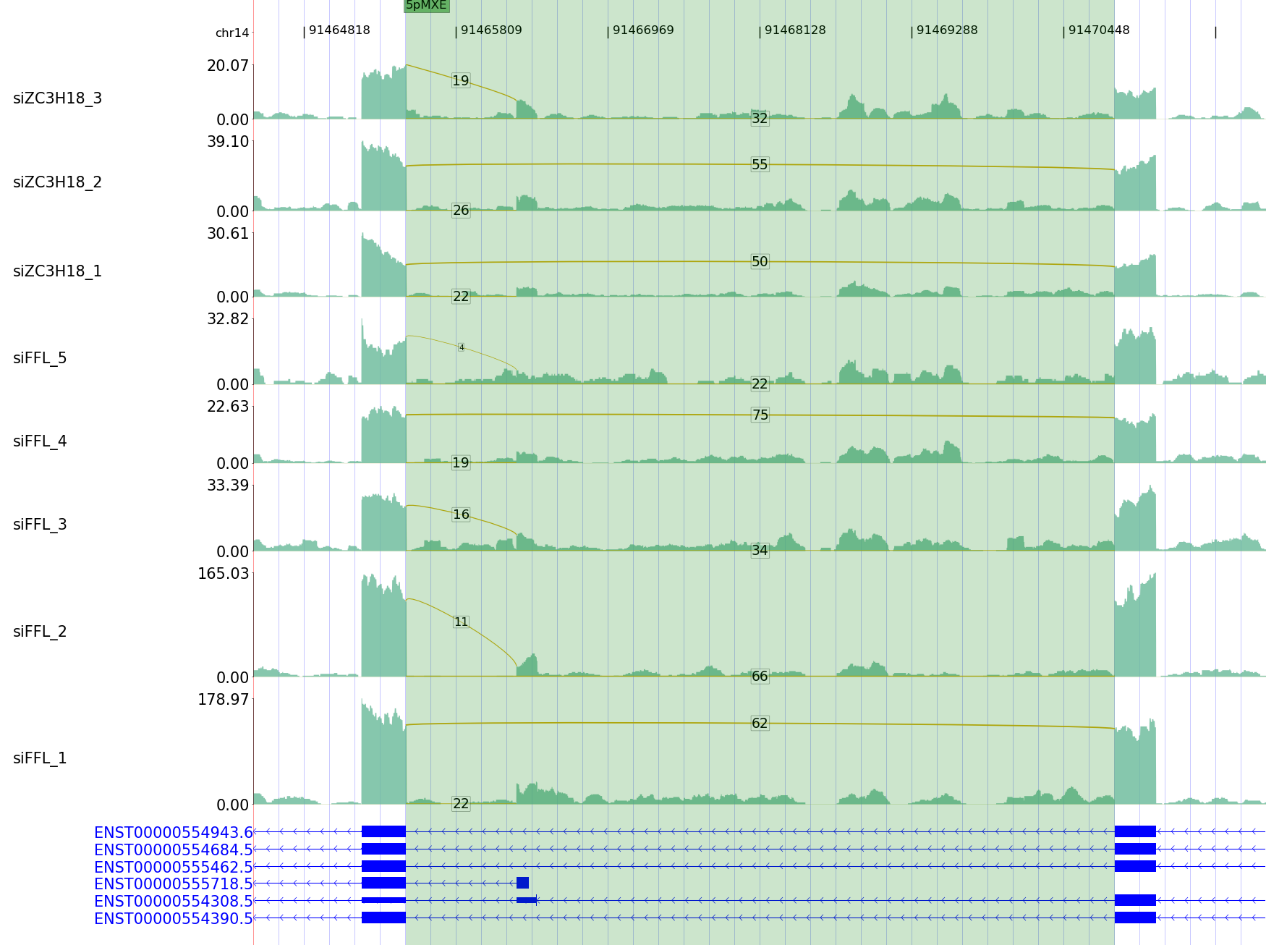

Supplement: Supplementary file 4 [file Table15.docx]
